# Supplementary material for: Effect of Aspergillus flavus Fungal Elicitor on the Production of Terpenoid Indole Alkaloids in Catharanthus roseus Cambial Meristematic Cells
Source: Molecules. 2018 Dec 11;23(12):3276. doi: 10.3390/molecules23123276 (PMC6320906; doi:10.3390/molecules23123276)
Supplement: Supplementary file 1 [file molecules-23-03276-s001.pdf]

molecules

Article

Effect of *Aspergillus flavus* fungal elicitor on the production of terpenoid indole alkaloids in *Catharanthus roseus* cambial meristematic cells

1. Supplementary Figures

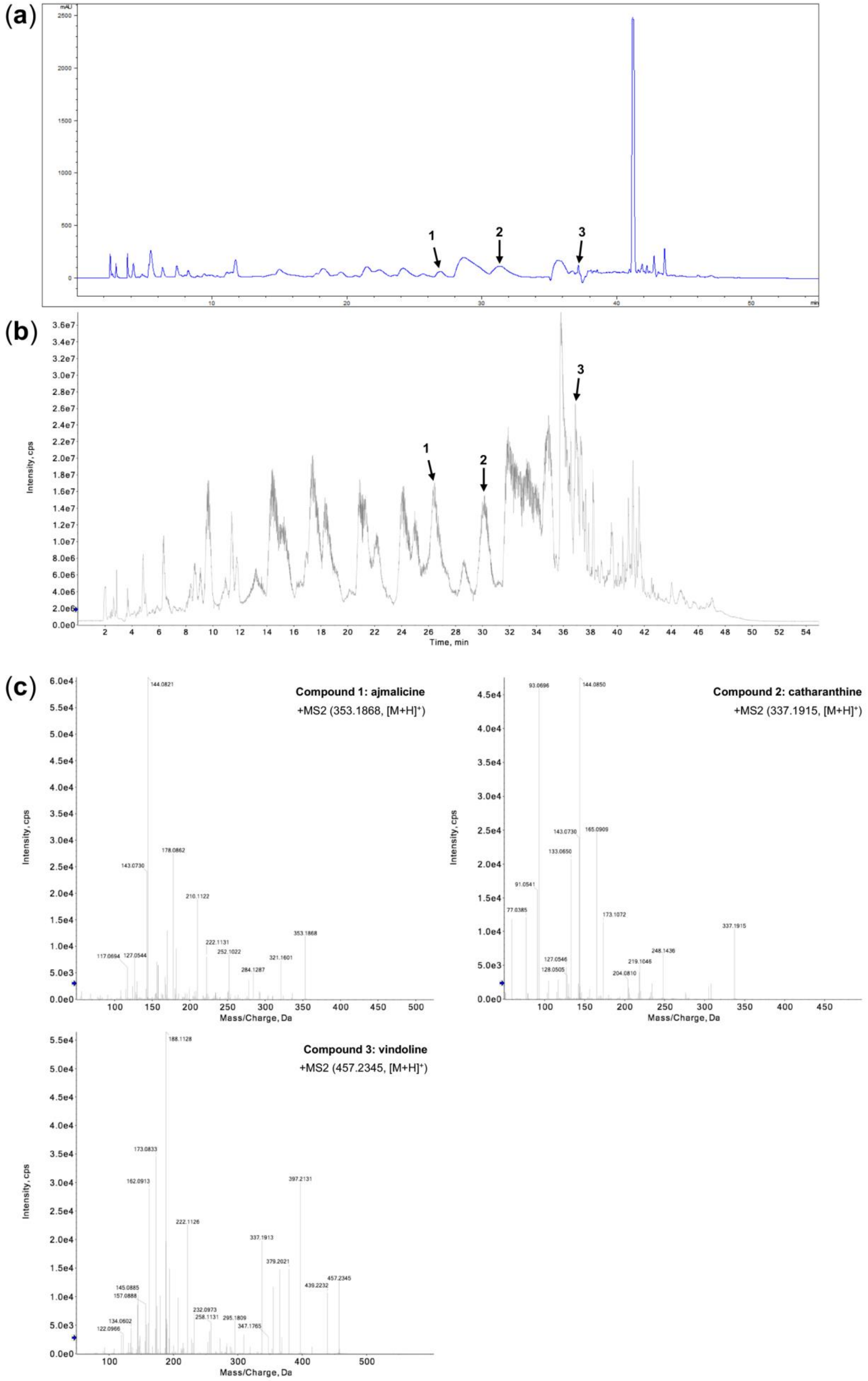
(a)
mAU
min
1
2
3
(b)
Intensity, cps
Time, min
1
2
3
(c)
Compound 1: ajmalicine
+MS2 (353.1868, [M+H]+)
144.0821
178.0862
143.0730
210.1122
222.1131
252.1022
353.1868
117.0694
127.0544
321.1601
284.1287
Intensity, cps
Mass/Charge, Da
Compound 2: catharanthine
+MS2 (337.1915, [M+H]+)
93.0696
144.0850
143.0730
165.0909
133.0650
91.0541
77.0385
173.1072
337.1915
248.1436
127.0546
128.0505
219.1046
204.0810
Intensity, cps
Mass/Charge, Da
Compound 3: vindoline
+MS2 (457.2345, [M+H]+)
188.1128
173.0833
162.0913
397.2131
222.1126
337.1913
379.2021
457.2345
439.2232
145.0885
157.0888
232.0973
258.1131
295.1809
134.0602
122.0966
347.1765
Intensity, cps
Mass/Charge, Da

Figure S1. HPLC-MS/MS spectra of the alkaloids in 6-day-old suspensions of *C. roseus* CMCs. (**a**) UV chromatogram at 280 nm; (1) ajmalicine; (2) catharanthine; (3) vindoline. (**b**) Total ion current (TIC) chromatogram. (**c**) MS spectra of the identified alkaloids.

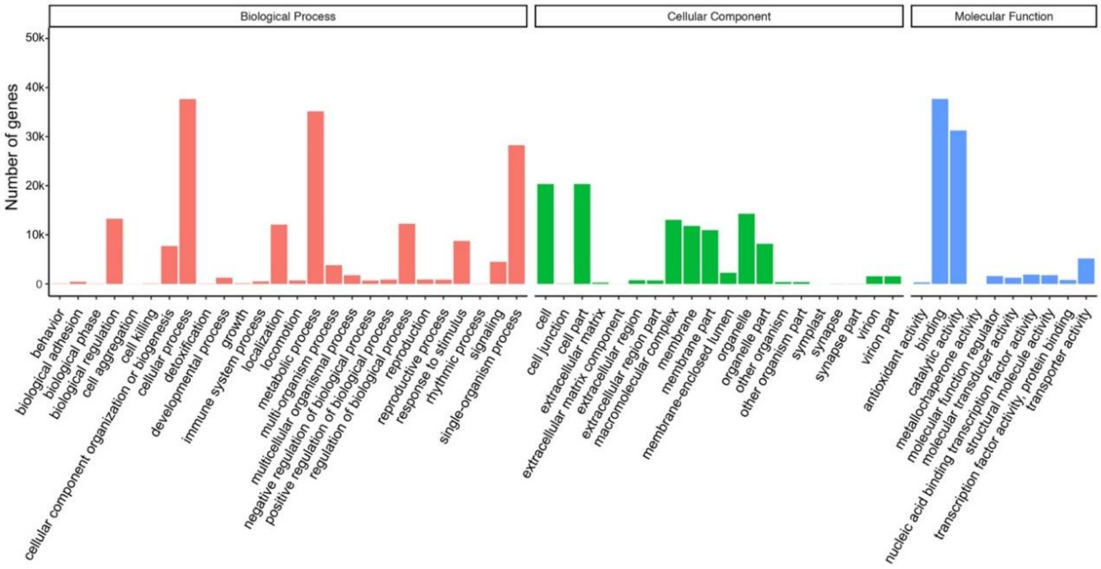

Figure S2. Gene Ontology (GO) functional classification of assembled unigenes. A total of 61,829 unigenes were assigned to at least one GO term and grouped into three main GO categories and 56 groups (26 groups in the "biological process" domain, 20 in the "cellular component" domain, and 10 in the "molecular function" domain). The y-axis indicates the number of genes in a sub-category.

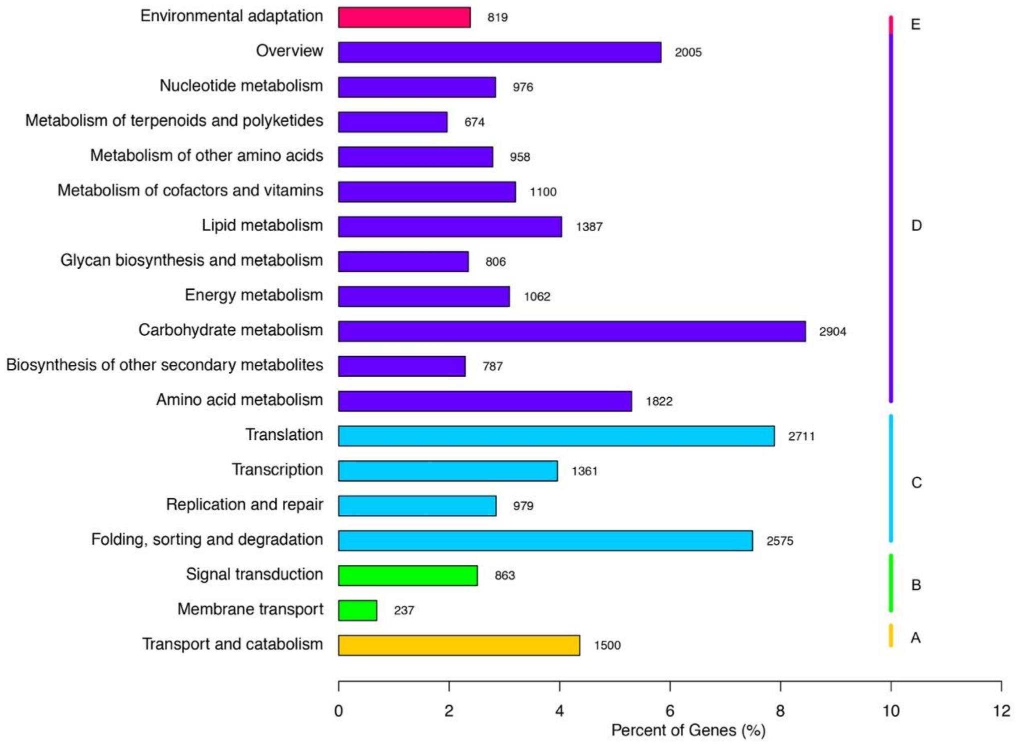

Figure S3. Functional classification and pathway assignment of assembled unigenes by Kyoto Encyclopedia of Genes and Genomes (KEGG). A total of 34,367 unigenes were classified to the five

main KEGG metabolic pathways: cellular processes (**A**), environmental information processing (**B**), genetic information processing (**C**), metabolism (**D**), and organismal systems (**E**). The y-axis represents the name of KEGG metabolic pathway. The x-axis indicated the number of unigenes annotated to the KEGG metabolic pathway and the ratio of their number to the total number of annotated unigenes.

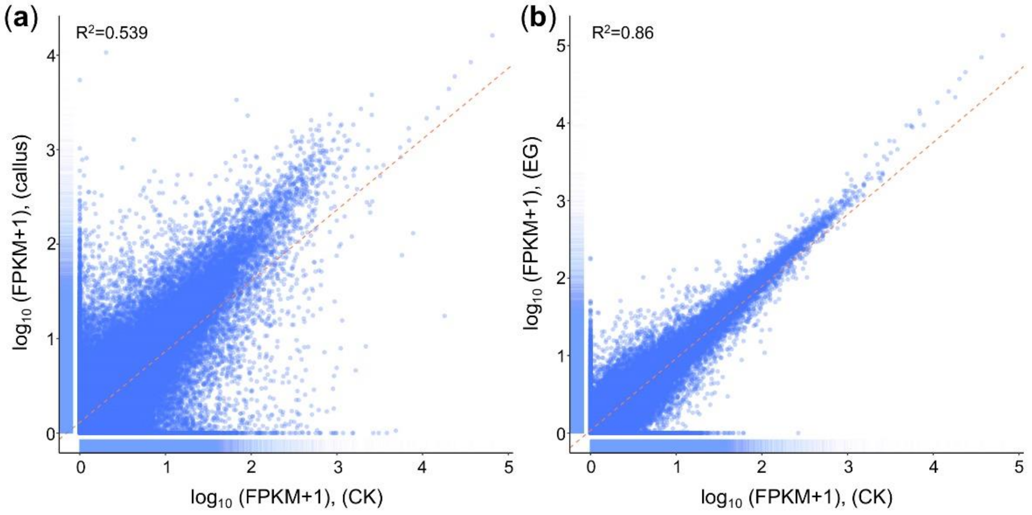

Figure S4. Correlation coefficient between the callus vs CK (**a**) and EG vs CK (**b**) samples. The x-axis indicated the $\log_{10}$ (FPKM+1) of the sample 1, the y-axis indicated the $\log_{10}$ (FPKM+1) of the sample 2, and R^2 was the square of the Pearson correlation coefficient.

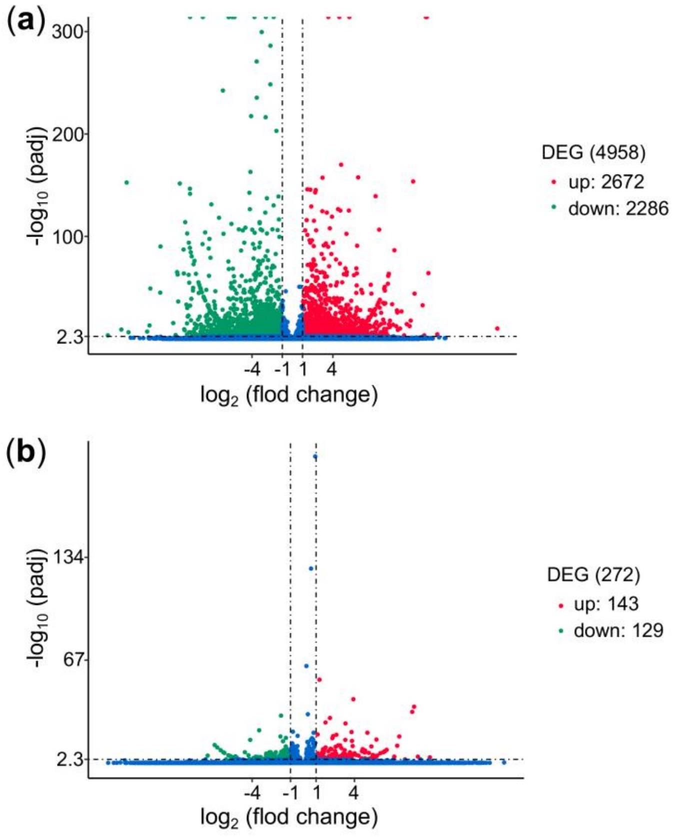

Figure S5. Correlation coefficient between the callus vs CK (**a**) and EG vs CK (**b**) samples. The x-axis indicated the log_{10} (FPKM+1) of the sample 1, the y-axis indicated the log_{10} (FPKM+1) of the sample 2, and R^2 was the square of the Pearson correlation coefficient.

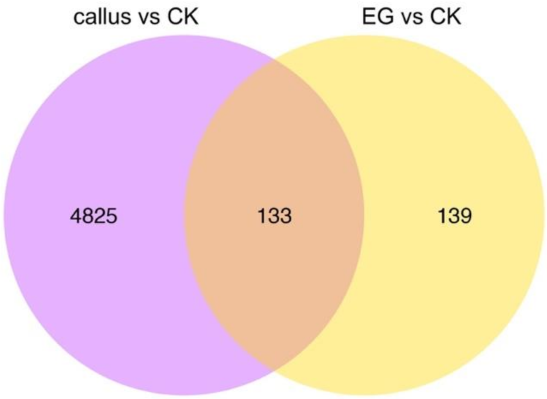

Figure S6. Venn diagram of DEGs from callus vs CK and EG vs CK samples.

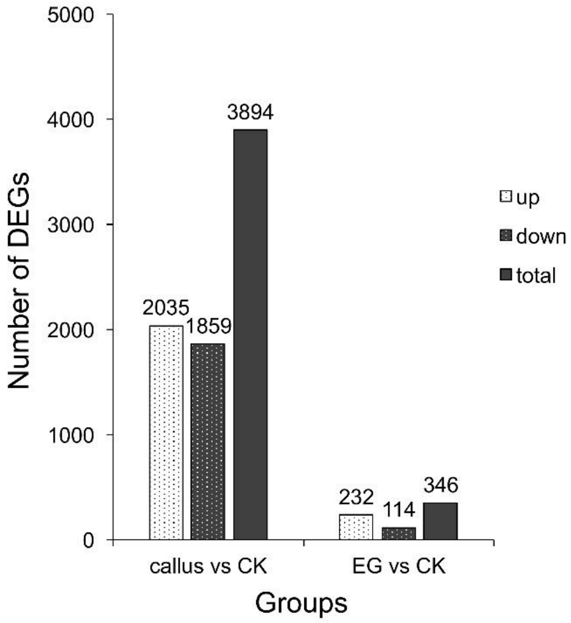

Figure S7. GO enrichment analysis of DEGs in callus vs CK and EG vs CK samples. The x-axis indicated the comparative combination, and the y-axis indicated the number of DEGs.

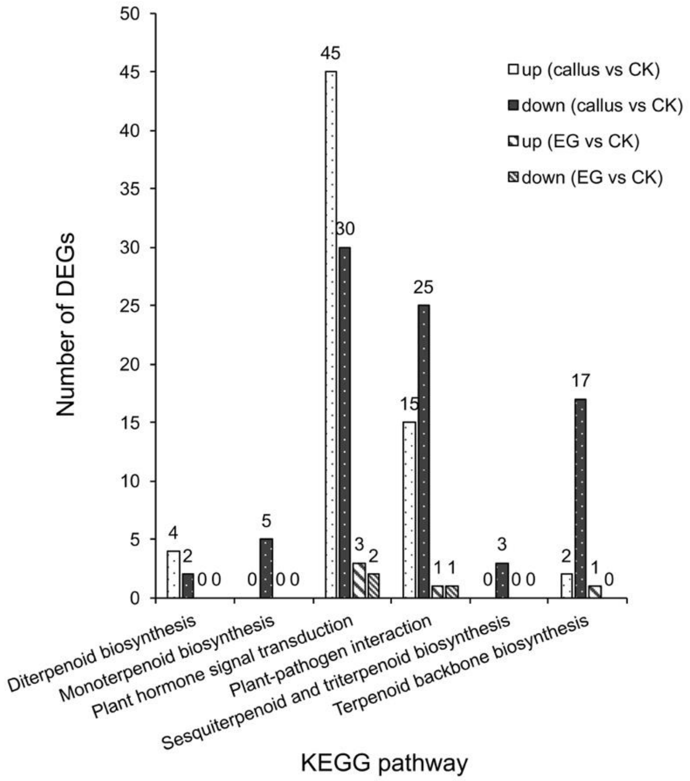

Figure S8. Partial results of KEGG pathway analysis of DEGs in callus vs CK and EG vs CK samples. The x-axis indicated the KEGG pathway, and the y-axis indicated the number of DEGs.

2. Supplementary Tables

Table S1. Summary of sequencing reads after filtering.

Sample	Raw Reads (n)	Clean reads (n)	Error (%) [1]	Q20 (%) [2]	Q30 (%) [3]	GC (%) [4]
callus	60,582,576	59,661,538	0.02	97.38	92.68	42.48
CK	51,466,966	50,694,970	0.02	97.28	92.60	42.80
EG	58,673,176	57,761,524	0.02	97.25	92.57	42.59

[1] Error (%): base error rate of clean reads; [2] Q20 (%): the percentage of bases with a Phred value above 20 in clean reads; [3] Q30 (%): the percentage of bases with a Phred value above 30 in clean reads; [4] GC (%): the percentage of G and C bases in clean reads.

Table S2. Summary of the sequence assembly results.

Type	Transcripts	Unigenes
Total Number (n)	121,532	105,552
Total Nucleotides (bp)	217,209,337	212,484,720
Min Length (bp)	201	201
Max Length (bp)	17,163	17,163
Mean Length (bp)	1,787	2,013
N50 (bp) [1]	2,845	2,892
N90 (bp) [2]	941	1072

[1] N50: a weighted median statistic in which 50 % of the total length is contained in unigenes greater than or equal to this value; [2] N90: a weighted median statistic in which 90 % of the total length is contained in unigenes greater than or equal to this value.

Table S3. Summary of functional annotation for assembled unigenes.

Database	Number of Unigenes	Percentage (%)
NR	79,711	75.51
NT	59,272	56.15
Swiss-Prot	63,496	60.15
Pfam	61,591	58.35
GO	61,829	58.57
KOG	26,674	25.27
KEGG	34,367	32.55
All annotated unigenes	83,742	79.33
Total unigenes	105,552	100

Table S4. Summary of KEGG pathways involved in the *Catharanthus roseus* transcriptome.

No.	Pathway Hierarchy 1	Pathway Hierarchy 2	KEGG Pathway	Pathway ID	Unigene Number
1	Cellular Processes	Transport and catabolism	Endocytosis	ko04144	608
2	Cellular Processes	Transport and catabolism	Peroxisome	ko04146	486
3	Cellular Processes	Transport and catabolism	Phagosome	ko04145	322
4	Cellular Processes	Transport and catabolism	Regulation of autophagy	ko04140	194
5	Environmental Information Processing	Membrane transport	ABC transporters	ko02010	237
6	Environmental Information Processing	Signal transduction	Phosphatidylino sitol signaling system	ko04070	291
7	Environmental Information Processing	Signal transduction	Plant hormone signal transduction	ko04075	572
8	Genetic Information Processing	Folding, sorting and degradation	Proteasome	ko03050	161
9	Genetic Information Processing	Folding, sorting and degradation	Protein export	ko03060	289
10	Genetic Information Processing	Folding, sorting and degradation	Protein processing in endoplasmic reticulum	ko04141	859

11	Genetic Information Processing	Folding, sorting and degradation	RNA degradation	ko03018	556
12	Genetic Information Processing	Folding, sorting and degradation	SNARE interactions in vesicular transport	ko04130	152
13	Genetic Information Processing	Folding, sorting and degradation	Sulfur relay system	ko04122	52
14	Genetic Information Processing	Folding, sorting and degradation	Ubiquitin mediated proteolysis	ko04120	713
15	Genetic Information Processing	Replication and repair	Base excision repair	ko03410	240
16	Genetic Information Processing	Replication and repair	DNA replication	ko03030	221
17	Genetic Information Processing	Replication and repair	Homologous recombination	ko03440	338
18	Genetic Information Processing	Replication and repair	Mismatch repair	ko03430	215
19	Genetic Information Processing	Replication and repair	Non-homologou s end-joining	ko03450	85
20	Genetic Information Processing	Replication and repair	Nucleotide excision repair	ko03420	304
21	Genetic Information Processing	Transcription	Basal transcription factors	ko03022	296
22	Genetic Information Processing	Transcription	RNA polymerase	ko03020	249
23	Genetic Information Processing	Transcription	Spliceosome	ko03040	816
24	Genetic Information Processing	Translation	Aminoacyl-tRN A biosynthesis	ko00970	393
25	Genetic Information Processing	Translation	RNA transport	ko03013	676
26	Genetic Information Processing	Translation	Ribosome	ko03010	741
27	Genetic Information Processing	Translation	Ribosome biogenesis in eukaryotes	ko03008	451

28	Genetic Information Processing	Translation	mRNA surveillance pathway	ko03015	588
29	Metabolism	Amino acid metabolism	Alanine, aspartate and glutamate metabolism	ko00250	223
30	Metabolism	Amino acid metabolism	Arginine and proline metabolism	ko00330	244
31	Metabolism	Amino acid metabolism	Arginine biosynthesis	ko00220	181
32	Metabolism	Amino acid metabolism	Cysteine and methionine metabolism	ko00270	308
33	Metabolism	Amino acid metabolism	Glycine, serine and threonine metabolism	ko00260	248
34	Metabolism	Amino acid metabolism	Histidine metabolism	ko00340	110
35	Metabolism	Amino acid metabolism	Lysine biosynthesis	ko00300	55
36	Metabolism	Amino acid metabolism	Lysine degradation	ko00310	137
37	Metabolism	Amino acid metabolism	Phenylalanine metabolism	ko00360	155
38	Metabolism	Amino acid metabolism	Phenylalanine, tyrosine and tryptophan biosynthesis	ko00400	246
39	Metabolism	Amino acid metabolism	Tryptophan metabolism	ko00380	107
40	Metabolism	Amino acid metabolism	Tyrosine metabolism	ko00350	180
41	Metabolism	Amino acid metabolism	Valine, leucine and isoleucine biosynthesis	ko00290	72
42	Metabolism	Amino acid metabolism	Valine, leucine and isoleucine degradation	ko00280	327
43	Metabolism	Biosynthesis of other secondary metabolites	Anthocyanin biosynthesis	ko00942	19
44	Metabolism	Biosynthesis of other secondary metabolites	Betalain biosynthesis	ko00965	3
45	Metabolism	Biosynthesis of other secondary metabolites	Caffeine metabolism	ko00232	6

46	Metabolism	Biosynthesis of other secondary metabolites	Flavone and flavonol biosynthesis	ko00944	13
47	Metabolism	Biosynthesis of other secondary metabolites	Flavonoid biosynthesis	ko00941	74
48	Metabolism	Biosynthesis of other secondary metabolites	Glucosinolate biosynthesis	ko00966	25
49	Metabolism	Biosynthesis of other secondary metabolites	Indole alkaloid biosynthesis	ko00901	7
50	Metabolism	Biosynthesis of other secondary metabolites	Isoflavonoid biosynthesis	ko00943	8
51	Metabolism	Biosynthesis of other secondary metabolites	Isoquinoline alkaloid biosynthesis	ko00950	93
52	Metabolism	Biosynthesis of other secondary metabolites	Monobactam biosynthesis	ko00261	49
53	Metabolism	Biosynthesis of other secondary metabolites	Phenylpropanoid biosynthesis	ko00940	518
54	Metabolism	Biosynthesis of other secondary metabolites	Stilbenoid, diarylheptanoid and gingerol biosynthesis	ko00945	50
55	Metabolism	Biosynthesis of other secondary metabolites	Tropane, piperidine and pyridine alkaloid biosynthesis	ko00960	79
56	Metabolism	Carbohydrate metabolism	Amino sugar and nucleotide sugar metabolism	ko00520	438
57	Metabolism	Carbohydrate metabolism	Ascorbate and aldarate metabolism	ko00053	190
58	Metabolism	Carbohydrate metabolism	Butanoate metabolism	ko00650	108
59	Metabolism	Carbohydrate metabolism	C5-Branched dibasic acid	ko00660	20

			metabolism		
60	Metabolism	Carbohydrate metabolism	Citrate cycle (TCA cycle)	ko00020	222
61	Metabolism	Carbohydrate metabolism	Fructose and mannose metabolism	ko00051	182
62	Metabolism	Carbohydrate metabolism	Galactose metabolism	ko00052	237
63	Metabolism	Carbohydrate metabolism	Glycolysis / Gluconeogenesi s	ko00010	479
64	Metabolism	Carbohydrate metabolism	Glyoxylate and dicarboxylate metabolism	ko00630	283
65	Metabolism	Carbohydrate metabolism	Inositol phosphate metabolism	ko00562	314
66	Metabolism	Carbohydrate metabolism	Pentose and glucuronate interconversions	ko00040	184
67	Metabolism	Carbohydrate metabolism	Pentose phosphate pathway	ko00030	208
68	Metabolism	Carbohydrate metabolism	Propanoate metabolism	ko00640	217
69	Metabolism	Carbohydrate metabolism	Pyruvate metabolism	ko00620	443
70	Metabolism	Carbohydrate metabolism	Starch and sucrose metabolism	ko00500	772
71	Metabolism	Energy metabolism	Carbon fixation in photosynthetic organisms	ko00710	287
72	Metabolism	Energy metabolism	Nitrogen metabolism	ko00910	113
73	Metabolism	Energy metabolism	Oxidative phosphorylation	ko00190	387
74	Metabolism	Energy metabolism	Photosynthesis	ko00195	107
75	Metabolism	Energy metabolism	Photosynthesis - antenna proteins	ko00196	33
76	Metabolism	Energy metabolism	Sulfur metabolism	ko00920	149
77	Metabolism	Glycan biosynthesis and metabolism	Glycosaminogly can degradation	ko00531	187
78	Metabolism	Glycan biosynthesis and	Glycosphingolip id biosynthesis - ganglio series	ko00604	40

		metabolism			
79	Metabolism	Glycan biosynthesis and metabolism	Glycosphingolipid biosynthesis - globo series	ko00603	36
80	Metabolism	Glycan biosynthesis and metabolism	Glycosylphosphatidylinositol(GPI)-anchor biosynthesis	ko00563	213
81	Metabolism	Glycan biosynthesis and metabolism	N-Glycan biosynthesis	ko00510	252
82	Metabolism	Glycan biosynthesis and metabolism	Other glycan degradation	ko00511	195
83	Metabolism	Glycan biosynthesis and metabolism	Other types of O-glycan biosynthesis	ko00514	14
84	Metabolism	Lipid metabolism	Arachidonic acid metabolism	ko00590	50
85	Metabolism	Lipid metabolism	Biosynthesis of unsaturated fatty acids	ko01040	104
86	Metabolism	Lipid metabolism	Cutin, suberine and wax biosynthesis	ko00073	30
87	Metabolism	Lipid metabolism	Ether lipid metabolism	ko00565	107
88	Metabolism	Lipid metabolism	Fatty acid biosynthesis	ko00061	144
89	Metabolism	Lipid metabolism	Fatty acid degradation	ko00071	238
90	Metabolism	Lipid metabolism	Fatty acid elongation	ko00062	77
91	Metabolism	Lipid metabolism	Glycerolipid metabolism	ko00561	270
92	Metabolism	Lipid metabolism	Glycerophospholipid metabolism	ko00564	415
93	Metabolism	Lipid metabolism	Linoleic acid metabolism	ko00591	55
94	Metabolism	Lipid metabolism	Sphingolipid metabolism	ko00600	160
95	Metabolism	Lipid metabolism	Steroid biosynthesis	ko00100	104
96	Metabolism	Lipid metabolism	Synthesis and degradation of ketone bodies	ko00072	55
97	Metabolism	Lipid	alpha-Linolenic	ko00592	130

		metabolism	acid metabolism		
98	Metabolism	Metabolism of cofactors and vitamins	Biotin metabolism	ko00780	91
99	Metabolism	Metabolism of cofactors and vitamins	Folate biosynthesis	ko00790	87
100	Metabolism	Metabolism of cofactors and vitamins	Lipoic acid metabolism	ko00785	17
101	Metabolism	Metabolism of cofactors and vitamins	Nicotinate and nicotinamide metabolism	ko00760	118
102	Metabolism	Metabolism of cofactors and vitamins	One carbon pool by folate	ko00670	68
103	Metabolism	Metabolism of cofactors and vitamins	Pantothenate and CoA biosynthesis	ko00770	160
104	Metabolism	Metabolism of cofactors and vitamins	Porphyrin and chlorophyll metabolism	ko00860	223
105	Metabolism	Metabolism of cofactors and vitamins	Riboflavin metabolism	ko00740	50
106	Metabolism	Metabolism of cofactors and vitamins	Thiamine metabolism	ko00730	54
107	Metabolism	Metabolism of cofactors and vitamins	Ubiquinone and other terpenoid-quinone biosynthesis	ko00130	176
108	Metabolism	Metabolism of cofactors and vitamins	Vitamin B6 metabolism	ko00750	65
109	Metabolism	Metabolism of other amino acids	Cyanoamino acid metabolism	ko00460	287
110	Metabolism	Metabolism of other amino acids	Glutathione metabolism	ko00480	354
111	Metabolism	Metabolism of other amino acids	Selenocompound metabolism	ko00450	100
112	Metabolism	Metabolism of other amino acids	Taurine and hypotaurine metabolism	ko00430	43
113	Metabolism	Metabolism of other amino acids	beta-Alanine metabolism	ko00410	244
114	Metabolism	Metabolism of terpenoids and	Brassinosteroid biosynthesis	ko00905	38

		polyketides			
115	Metabolism	Metabolism of terpenoids and polyketides	Carotenoid biosynthesis	ko00906	167
116	Metabolism	Metabolism of terpenoids and polyketides	Diterpenoid biosynthesis	ko00904	78
117	Metabolism	Metabolism of terpenoids and polyketides	Limonene and pinene degradation	ko00903	40
118	Metabolism	Metabolism of terpenoids and polyketides	Monoterpenoid biosynthesis	ko00902	43
119	Metabolism	Metabolism of terpenoids and polyketides	Sesquiterpenoid and triterpenoid biosynthesis	ko00909	35
120	Metabolism	Metabolism of terpenoids and polyketides	Terpenoid backbone biosynthesis	ko00900	314
121	Metabolism	Metabolism of terpenoids and polyketides	Zeatin biosynthesis	ko00908	80
122	Metabolism	Nucleotide metabolism	Purine metabolism	ko00230	798
123	Metabolism	Nucleotide metabolism	Pyrimidine metabolism	ko00240	629
124	Metabolism	Overview	2-Oxocarboxylic acid metabolism	ko01210	238
125	Metabolism	Overview	Biosynthesis of amino acids	ko01230	1011
126	Metabolism	Overview	Carbon metabolism	ko01200	1156
127	Metabolism	Overview	Degradation of aromatic compounds	ko01220	42
128	Metabolism	Overview	Fatty acid metabolism	ko01212	300
129	Organismal Systems	Environmental adaptation	Circadian rhythm - plant	ko04712	168
130	Organismal Systems	Environmental adaptation	Plant-pathogen interaction	ko04626	651

Table S5. Gene transcription factor analysis of unigenes.

Gene ID	Family
Cluster-10157.0_2	Orphans
Cluster-10157.1_1	Orphans
Cluster-1049.0_1	AP2-EREBP
Cluster-10773.4_0	SET

Table S6. Sequences of primers for *Catharanthus roseus* genes used in qRT-PCR assay.

Gene	Sequence of F/R primer (5′- 3′)
D4H (U71605)	F: TACCCTGCATGCCCTCAACC<br>R: TTGAAGGCCGCCAATTTGAT
G10H (AJ251269)	F: TGAATGCTTGGGCAATTGGA<br>R: GCAAATTCTTCGGCCAGCAC
GES (JN882024)	F: TTGTTTTCGATTGCTTCG<br>R: TCTATGTCTTGGTTGCTCTA
IRS (KF561460)	F: CCTAGGCTAAATGTCCCAAA<br>R: GTCTATGGACAGACCATGTT
LAMT (EU057974)	F: GAGTAATTGATGCAGCCAAG<br>R: TTGATTGGATCAAAGATTGG
ORCA3 (AJ251250)	F: CGAATTCAATGGCGGAAAGC<br>R: CCTTATCTCCGCCGCGAACT
RPS9 (AJ749993)	F: TGAAGCCCTTTTGAGGAGGATG<br>R: TGCCATCCCAGACTTGAAAACA
SGD (EU072423)	F: ATGAGAGCTCTTGTAGGAAGCCGT<br>R: GCGCACTTCCTTCCCATCAACTTT
STR (X61932)	F: TGACAGTCCCGAAGGTGTGG<br>R: CGCCGGGAACATGTAGCTCT
TDC (M25151)	F: TCCGAAAACAAGCCCATCGT<br>R: AAGGAGCGGTTTCGGGGATA
